# Spatial Exploration Behavior – A Novel Cognitive Marker for Alzheimer’s Disease?

**DOI:** 10.1002/alz.086213

**Published:** 2025-01-03

**Authors:** Vaisakh Puthusseryppady, David L Sultzer, Elizabeth R Chrastil

**Affiliations:** ^1^ University of California Irvine, Irvine, CA USA; ^2^ The UC Irvine Institute for Memory Impairments and Neurological Disorders (UCI MIND), Irvine, CA USA

## Abstract

**Background:**

Alterations to spatial navigation have been suggested by previous studies to represent an early cognitive marker for those with and at risk of Alzheimer’s Disease (AD). However, with most of these studies focusing on spatial memory (usage of formed spatial representations), very little is known about the extent to which spatial exploration (process by which spatial representations are formed) may be altered in AD. The aim of this study is to investigate how spatial exploration behavior may be altered in individuals with and at risk of AD, and the extent to which individuals can be classified into their clinical status based on their exploration behavior.

**Method:**

10 cognitively unimpaired older adults, 10 adults with mild cognitive impairment (MCI), and 10 adults with clinical AD are recruited (all participants aged > 50 years). All participants undergo a Virtual Supermarket Learning Task using immersive walking virtual reality to measure their exploration behavior and spatial memory. In the exploration phase, participants freely explore a 13×6‐meter virtual supermarket for 7 minutes and learn the locations of 6 floating target objects. Here, various exploration behaviors (distance travelled, turning angles, object visits, etc.) are measured. In the wayfinding phase, participants are instructed to navigate from one target object to another, without feedback, and wayfinding success (proportion of correct trials) is measured. In the pointing phase, participants are instructed to point from one target object to another, without feedback, and angular error (deviation from correct location) is measured.

**Result:**

Performance of the AD and MCI groups in all task phases are compared to that of the healthy controls to assess alterations to exploration behavior as well as wayfinding and pointing performance. Furthermore, we assess how well we can classify the AD and MCI groups from controls using their exploration behavior, and compare this with the classification accuracy obtained from using their wayfinding/pointing performance.

**Conclusion:**

The study results will highlight the extent to which spatial exploration behavior can be considered a novel and sensitive cognitive marker for AD, having important implications for understanding early cognitive changes in AD and potentially identifying candidates for therapeutic intervention.

